# Changes of oxidative stress in 3D film to be prepared for echocardiography: A randomized controlled study

**DOI:** 10.1097/MD.0000000000039419

**Published:** 2024-08-30

**Authors:** Emel Demir, Vesile Duzguner, Nuh Yilmaz, Erhan Yengil

**Affiliations:** a Children Health and Diseases Nursing Department, Faculty of Health Sciences, Hatay Mustafa Kemal University, Hatay, Turkey; b Emergency and Disaster Management Department, Faculty of Health Sciences, Ardahan University, Ardahan, Turkey; c Department of Pediatric Cardiology, Faculty of Medicine, Hatay Mustafa Kemal University, Tayfur Ata Sökmen, Hatay, Turkey; d Department of Family Medicine, Faculty of Medicine, Hatay Mustafa Kemal University, Tayfur Ata Sökmen, Hatay, Turkey.

**Keywords:** 3D film, echocardiography, oxidative stress

## Abstract

**Background:**

Echocardiography (ECHO) is a nonprocedure that causes acute stress in children. Fear, anxiety, and fluctuations in children’s blood pressure and heart rate can potentially lead to alterations in echocardiographic measurements. The insufficient research on virtual reality pediatric preparation applications, particularly in the context of echocardiographic procedures, underscores the necessity for additional studies focusing on pediatric patients. This study sought to assess the impact of virtual reality applications tailored explicitly for ECHO on children’s oxidative stress levels.

**Methods:**

This was a prospective, randomized, controlled experimental study. Forty-eight children (experimental/24, control/24) aged 7 to 12 years who had an ECHO appointment in the pediatric cardiology outpatient clinic in the 3 months from June to August 2019 participated in the study. Participants whose blood analyses showed hemolysis were eliminated, and the study was completed with 37 children in total: 16 children in the experiment and 21 children in the control. Post hoc power analysis was performed for sample adequacy, and the power of the study was found to be 0.99. A 3D film was prepared for the research and applied to the experimental group before the procedure. All children’s oxidative stress levels (cortisol, malondialdehyde, nitric oxide) and glutathione levels were checked after ECHO.

**Results:**

The stress hormone cortisol and malondialdehyde levels were lower in the 3D-applied experimental group than in the control group. As another crucial anti-stress antioxidant factor, glutathione level increased in the experimental group compared to the control group (*P* < .05).

**Conclusion:**

The research revealed that the 3D film used to prepare ECHO reduces the stress parameters associated with heart risk and may affect the ECHO measurements. At the same time, the study also proved the positive effect of 3D film preparation by increasing the anti-stress factor.

## 1. Introduction

Echocardiography (ECHO) is a noninvasive procedure used to diagnose pediatric cardiology in outpatient clinics and is feared by pediatric patients.^[1]^ At the same time, ECHO is essential in diagnosing heart diseases, including simple symptoms. Simple symptoms, including overlooked murmur, are evaluated with this method, bringing the diagnostic reliability of pediatric cardiologists closer to 100%.^[2]^ ECHO, which includes large-screen monitors and sounds, is performed in a particular room with a special device, and preparation for the procedure is often overlooked because it differs from routine medical procedures. However, the child’s fear and stress, blood pressure, and heart rate changes may cause changes in echocardiographic measurements.

For this reason, sedation may be required when this stressful procedure is applied to some children. Medications administered during sedation range from minor to significant symptoms requiring intervention, in which cardiac and respiratory depression are suppressed. Therefore, against these risks, the pediatric patient should be monitored regarding oxygen saturation, heart rate, and blood pressure. Although it seems like a simple diagnostic technique, when sedation is required, it also exposes the child to additional procedures such as time, personnel, and monitoring regarding risks and costs.^[1,3]^ Preparing children by reducing their fear of this procedure can reduce the need for sedation and reduce the frequency of risks associated with the sedation procedure.

It may increase the child’s symptoms by triggering acutely developing stress hormones in children at risk of heart disease and present due to diagnosis.^[4]^ Concomitant effects on cardiovascular, mental, and metabolic symptoms have been reported in children with ongoing chronic stress.^[5,6]^ In potentially harmful and uncontrollable situations, the human body uses 2 basic mechanisms in response to stress. These release stress hormones through activating the autonomic nervous system and hypothalamus–pituitary–adrenal axis.^[5,7]^ The body responds to acute stress using physiological and behavioral responses such as heart rate. In the laboratory environment, the hormonal values of stress in the blood can be determined by the oxidative responses in the cell.^[5]^ Cortisol is a vital stress response marker associated with most cardiovascular diseases.^[4]^ Oxidative stress is a physiopathological condition associated with deterioration in the balance of prooxidant-antioxidant capacity, which can potentially cause neuronal damage in favor of prooxidant capacity. Brain cells are sensitive to ROS production. Because the brain uses most of the body’s total oxygen, and the brain’s antioxidant capacity is limited. In cases where free oxygen radicals increase, and antioxidant capacity is insufficient, oxidative stress negatively affects cell functions by directly damaging cell proteins, deoxyribonucleic acid (DNA), and lipids.^[8]^ There is a positive correlation between cortisol levels and a high risk of cardiovascular diseases.^[4]^ The hormonal and then cellular effects of stress may also affect the course of the disease by revealing new symptoms unrelated to the child’s disease.

If coping mechanisms for acute stress reactions are activated, adverse effects in children and adolescents can be prevented. In cases of acute stress with a defined cause, such as during childhood medical procedures, it can be resolved using some techniques [trier social stress test (TSTT), child life specialists (CLS)] and methods (VR, Phone).^[5,9,10]^ The literature reports that infants and children listen to music, watch something on the phone, and watch cartoons, and kaleidoscopes are used during the procedure.^[1]^ These procedures aim to reduce fear and anxiety by diverting the child’s attention and do not include preparation for the procedure. Research on watching cartoons using magnetic resonance imaging (MRI), computerized tomography (CT), and ultrasound footage shows that fear and anxiety are reduced during the procedure.^[10]^ Today, VR techniques are compatible with are compatible with nursing education, patient simulations, and procedure preparation.

Moreover, procedures prepared with VR techniques provide users with standard training and preparation opportunities and can serve many users for the same process.^[11,12]^ The lack of research on VR pediatric preparation applications, including ECHO procedures, reveals the need for such studies in patients. Due to its cost and ease of application, virtual reality (VR) may be a suitable alternative for the sedation procedure, which is not recommended unless necessary against its risks.

This study aims to determine the effect of 3D film prepared for ECHO on oxidative stress changes that display stress symptoms in children.

## 2. Methods

### 2.1. Study design and sample

The research is a prospective randomized controlled experimental study. The research was conducted in a cross-sectional type using the whole count method. It was conducted with children aged 7 to 12 who came for ECHO in a university hospital and pediatric cardiology outpatient clinic. The cross-sectional time of the study covers 3 months from June to August 2019, which was chosen as the period when ECHO appointments were intense, which was the school holiday. By randomization, 48 children (experimental/24, control/24) were included in the study during this period. Blood samples from the laboratory for the study were excluded because 8 blood samples in the hemolyzed experimental group and 3 in the control group were unsuitable for biochemical analysis. The study was completed with 37 children, 16 in the experiment and 21 in the control. The study was designed with a randomized trial according to the CONSORT 2010 checklist.

### 2.2. Sample size

At the end of the study, post hoc power analysis was performed for the sample size. According to the nitric oxide (NO) mean and standard deviation values of the control and experimental groups, the power of the study was found to be 0.99, with an effect size of 2 and an alpha margin of error of 0.05. The sample size was sufficient according to these values.

### 2.3. Inclusion criteria and exclusion criteria

Inclusion criteria for the study: 7 to 12 years of age; suspicion or presence of acenetic heart disease in the disease group; having an appointment in the pediatric cardiology outpatient clinic for ECHO; voluntary consent of parents and children to participate in the research.

Exclusion criteria: cyanotic heart disease in children; indication of cardiac surgery in the pre-post-op period; children who do not agree to participate in the study and who are older and younger than the age range.

### 2.4. Data collection

#### 2.4.1. Enrolment and allocation

In the study, the researcher identified patients for whom the pediatric cardiologist had previously made an awppointment for ECHO. The researcher took hemogram blood routinely taken from the children who came for ECHO from the laboratory (called E). In this hemogram blood sample, oxidative stress levels will be checked for all groups.

At this stage, researcher E took blood daily, centrifuged it, kept it in a refrigerator at −22 °C for 1 day, and stored it at −80 °C until the sample collection period was completed for biochemical analysis. Eleven cases whose hemogram contained insufficient or hemolyzed plasma were eliminated (Experiment n:8, Control n:3) (CONSORT flow diagram).

#### 2.4.2. Randomization

The computer secretary who registered the ECHO appointment randomly assigned the children. A bag containing 48 red and white cards was created at the beginning of the research and given to the journal secretary. During an ECHO appointment, a child was tasked with selecting cards from a group and distributing them randomly between experimental and control groups.^[13]^ Patients admitted to the outpatient clinic over 3 months were randomly assigned to either the experimental group (identified by red markers) or the control group (identified by white markers). The research was designed in a double-blind manner. The children participating in the study were unaware of which group they were assigned to. The nurse who took the blood, the researcher who did the ECHO of the children, and the researcher who did the biochemical analyses did not know the experimental and control groups. Researcher E, who randomized and administered the intervention after randomization, knew the experimental and control groups.

#### 2.4.3. Intervention

The group was determined according to the randomization of the mother and child who entered the polyclinic during registration. After this randomization, researcher E received sociodemographic information from the mother in a room separate from ECHO in the outpatient clinic at the first encounter.

The ECHO procedure was prepared using 3D film for the experimental group of children with red cards. The researcher applied VR film E to the children in the experimental group (n = 24). The researcher introduced VR glasses to the child and discussed the movie’s content. The researcher made the child sit in the portable chair and put VR glasses on the child under the mother’s supervision. The film was watched in a particular room reserved in the polyclinic while the researcher and his/her mother were with him/her. The child was told he/she could remove the glasses if he/she did not want to go on. After watching the movie, the glasses were removed, and we waited 3 minutes. Immediately afterward, he/she was taken to the Pediatric ECHO room, and the researcher’s doctor took his/her ECHO. Then, blood from the children was sent to the laboratory for routine blood collection. Blood collection was done by nurses in the pediatric blood collection department on the same floor.

In the study, the same nurse performed the entire blood collection procedure, and the nurse did not know the experimental control groups before drawing blood. At this stage, Researcher E centrifuged the blood and kept it in the refrigerator at −22 °C for 1 day, then stored it at −80 °C until the sample collection period was completed for biochemical analysis.

The control group (n = 24) of children with a white card was verbally prepared for ECHO by researcher E in the outpatient clinic according to routine hospital procedures. Then, the child, who was taken to the ECHO room and whose ECHO was taken by the researcher doctor, was sent to the laboratory for routine blood collection. For analysis, researcher E took this blood from the laboratory at the end of the process, centrifuged, and stored it in a −22 °C refrigerator for 1 day and at −80 °C until the sample collection period was completed.

#### 2.4.4. *3D* ECHO *film content*

**Figure 1.**
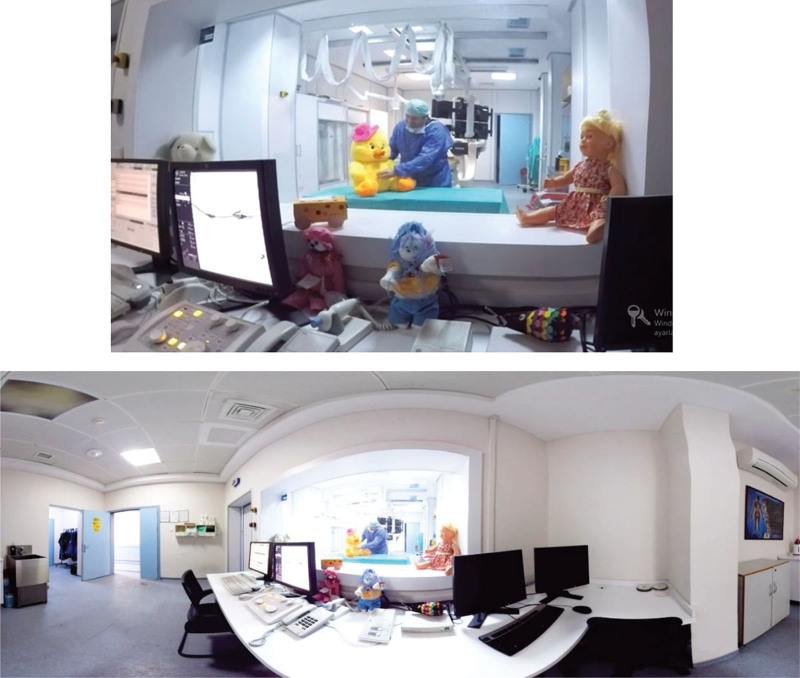
Part of 3D echocardiography film.

This movie was shot in the University hospital, with the child’s entrance and the health personnel he would encounter. The places he would see in the hospital were introduced to the child with colorful, musical, and toy decorations. In the ECHO room, the doctor who would perform the procedure demonstrated it on a large toy (Fig. 1).

### 2.5. Outcomes

Sociodemographic form (Children’s age, gender, and parents’ age) was used in the research. Stress and antioxidant levels in both groups before and after the ECHO were evaluated using statistical analysis methods. Both groups were statistically assessed between themselves and after analyzing the stress levels in the blood biochemically after the procedure.

### 2.6. Data analysis

Oxidative stress levels will be evaluated for both groups (16 experiments, 21 controls). At this stage, the biochemist controlled the preoperative blood. In experiment 8, control 3, blood with insufficient or bloody serum samples was excluded from the study. The experimental group 16 and control group 21 were determined to be suitable for analysis for all groups. The researcher biochemist performing the analyses will not know the experiment and control group (CONSORT flow diagram).

#### 2.6.1. Biochemical analyses

The blood samples were centrifuged at 3000 rpm for 10 minutes at room temperature. After centrifugation, the samples were stored in a refrigerator at −22 °C for 1 day and then transferred to a −80 °C freezer until testing.

To measure glutathione (GSH) levels, a commercial enzyme-linked immunosorbet assay (ELISA) kit (Cayman Chemical, Catalog No. 703002) was used, following the method described by Sedlak and Lindsay.^[14]^ The results of GSH levels were reported in micromoles (μM).

For the analysis of NO concentration in plasma samples, a commercial ELISA kit (Cayman Chemical, Catalog No. 780001) was used. This kit indirectly measures the nitrite levels in the samples based on the Griess reaction. The results of NO concentration were expressed in μM. To determine lipid peroxidation levels, the malondialdehyde (MDA) concentration in plasma samples was measured using a commercial kit (Cayman Chemical, Catalog No. 10009055). MDA was a marker of lipid peroxidation, and its concentration is reported in μM. The cortisol level, which indicates oxidative stress, was measured in plasma using a commercial kit from DRG (Catalog No. EIA-1887).

These assays and measurements were conducted to assess various oxidative stress markers in the blood samples, including NO, lipid peroxidation, cortisol levels, and antioxidant GSH levels.

#### 2.6.2. Statistical analyses

Statistical analyses were performed using the SPSS 1-way ANOVA procedure (Statistical Package for the Social Sciences; 25.0). The data were presented as mean ± standard error (S.E.). Statistical significance was determined based on *P* values, with values < .05 considered statistically significant.

### 2.7. Ethical considerations

This study was conducted following the approval of an Ethical Board Committee at a university (Approval No. 09.01.2019-N0:06). Before the research, both the children and their mothers were fully informed about the purpose and procedures of the study. The mothers provided their consent to participate, which was obtained verbally and in writing.

## 3. Results

### 3.1. Baseline characteristics

According to the sociodemographic data, the maternal age is normally distributed (0.01), and the father’s age is normally distributed (0.05) in the distribution of the experimental and control groups. The distribution of maternal and paternal age in the experimental and control groups was homogeneous, and the difference in the averages was not statistically significant. The age and gender distribution of the children was homogeneous compared to the experimental and control groups, and the difference was not statistically significant (*P* < .05) (Table 1).

**Table 1:** Sociodemographic data.

Characteristics	Virtual reality group (n = 16)	Control group (n = 21)	*P*
Child gender, number (%)			
Boy	7 (43.7)	9 (42.8)	.957
Girl	9 (56.2)	12 (57.1)	
Child age (7–16 year), mean ± SD	13.56 ± 2.52	12.23 ± 3.65	.368
Mother age, mean ± SD	39 ± 5.50	37.05 ± 5.97	.944
Father age, mean ± SD	42.63 ± 6.75	39.81 ± 6.92	.915

*P* < .05, Mann–Whitney *U* test.

### 3.2. Oxidative stress and antioxidative changes

NO levels were 3.31 ± 0.12 in the control group and 3.09 ± 0.09 in the VR-ECHO group. However, despite a decrease, it does not mean statistical significance (*P* > .05; data not shown).

**Figure 2.**
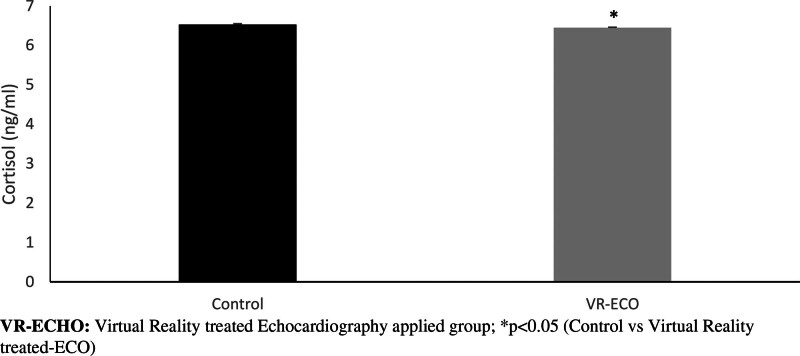
Changes in cortisol levels.

Oxidative stress hormone cortisol level (Fig. 2) was significantly decreased in the VR-ECHO group compared to the control group (*P* < .05).

**Figure 3.**
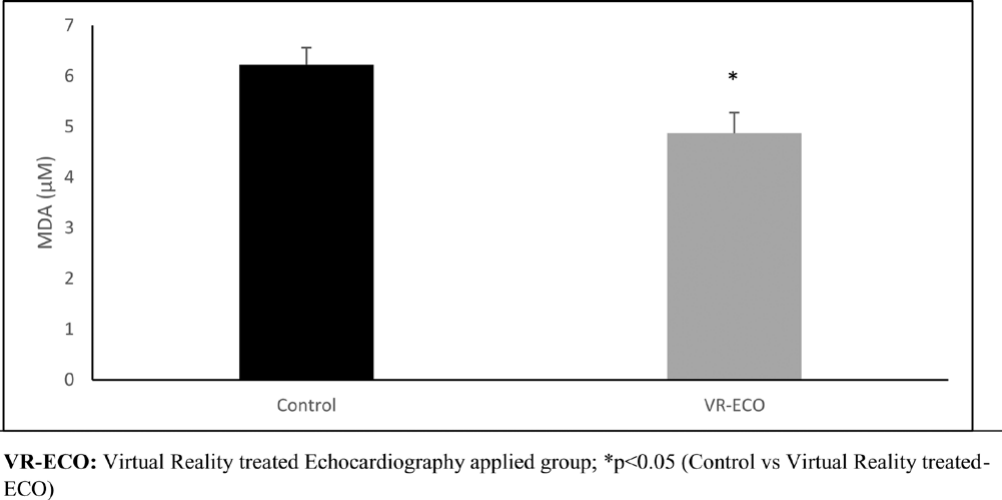
Changes in malondialdehyde levels.

VR application contributed to the statistically significant improvement of MDA levels (Fig. 3).

**Figure 4.**
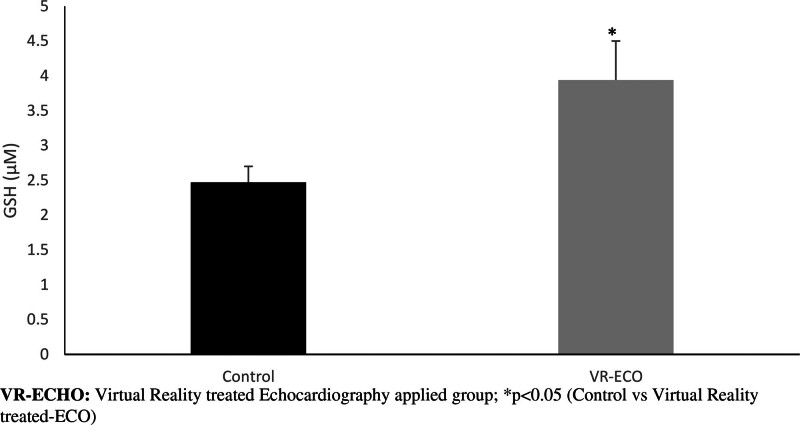
Changes in glutathione levels.

As another critical antioxidant factor, the GSH level increased in the VR-ECHO group compared to the control group (*P* < .05) (Fig. 4).

## 4. Discussion

The research revealed the oxidative stress changes of children prepared for ECHO with 3D film. The study, which examined 877 effective studies in pediatric cardiology between 1980 and 2020, extracted the characteristics of the researchers in this field. Congenital heart diseases and biomarkers are the most common research areas in pediatric cardiology. According to the research content, the need for studies in which biomarkers containing oxidative hormones are measured in future studies has been stated.^[15]^ The absence of ECHO preparation in 100 effective studies during the period under review may contribute to the literature with the oxidative stress data in the ECHO of our research. Pediatric cardiology emphasizes that in tests other than ECHO, some symptoms can be ignored in 10% of the cases. A study conducted with 3692 cases at the age of 7 reported that diagnostic tests had a margin of error of about 6% in evaluating clinical findings. In the methods used by the pediatric cardiologist, the initial diagnosis (clinical) and the final diagnosis (clinical + laboratory tests), there is 90% diagnostic compatibility for cases where ECHO is not performed.^[2]^ Considering that ECHO is an important diagnostic measure in the definitive diagnosis of pediatric cardiologists, effective methods must be transformed into routine practices in preparing this procedure.

According to our results, cortisol and MDA, which are oxidative stress markers, increase in children undergoing ECHO. Increased stress responses were lower in children in the experimental group who received VR-ECHO compared to the control group.

It is known that children with congenital heart disease are susceptible to the effects of oxidative stress. It has been determined that MDA values increase in the cyanotic group, especially in cyanotic heart diseases.^[16]^ Nahar et al^[17]^ found high MDA values in their study of patients with panic disorder and found that antioxidant vitamins and GSH values were lower than in control patients. In a different study that studied major depression and general anxiety disorder, it was reported that oxidative stress increased. In the same study, it was emphasized that oxidative stress can affect the development of depression in various ways and that this may occur through structural and proportional disorders of polyunsaturated fatty acids, excessive production of cytokines, decrease in the amount of catecholamines, deterioration in the density and functions of serotonergic and catecholaminergic receptors, and decrease in the catecholamine binding sites of the receptor.^[18]^ Ercan et al^[19]^ evaluated acenetic and cyanotic heart disease oxidative stress states with total antioxidant capacity (TAC), total oxidant capacity (TOC), and oxidative stress index (OSI). It has been reported that oxidative stress values in acynetic patients are close to normal.^[19]^ In our research results, oxidative stress values were reported to be close to those of regular patients, and it was done in the acenetic heart disease group, which excluded the negative effect of cyanotic heart findings, which affected oxidative stress.

The association between psychophysiological stress and the up regulation of nitricoxide synthase (NOS) mRNA expression, alongside increased enzymatic activity, is well-documented. Elevated NO levels generated during stress disrupt various components and fundamental stress response mechanisms, harming cellular components, including proteins, lipids, and DNA. This disruption can precipitate several stress-induced secondary neurological disorders, such as anxiety, depression, and cognitive deficits. In a recent study, VR treatment was employed as an intervention to reduce NO levels. Although a reduction in NO levels was observed following VR treatment, this decrease did not reach statistical significance. This result suggests that while VR treatment may have a potential role in modulating NO levels, its effect was not strong enough to be conclusive in this study. Further research with larger sample sizes and more rigorous methodologies may be needed to establish the effectiveness of VR treatment in reducing NO levels and its subsequent impact on stress-related neurological disorders.^[20]^

At the same time, the positive effects of VR in ECHO preparation were evaluated by measuring these changes with specific GSH values that show the antioxidant capacity of pediatric patients. Temel et al^[21]^ examined the antioxidant reserve capacities in acyonotic and cyanotic congenital heart diseases. According to the research results, there is a positive correlation between the age of the children and their preoperative antioxidant capacity. Hadley et al^[22]^ evaluated the oxidative stress responses of children after cardiac surgery. It has been stated that oxidative stress responses are significant in morbidity rates in the postoperative period. Likewise, the research findings indicated that patients exhibited heightened antioxidant system responses as their age increased.^[22]^ Satriano et al^[23]^ examined GSH levels in the preoperative period in patients undergoing open heart surgery. It has been stated that the increase in GSH levels in the perioperative period is significant as a mechanism compensating for the oxidative damage that develops due to complications during the surgical procedure. According to our research results, the VR-ECHO application has determined that it increases the level of GSH, which has many important cellular and extracellular antioxidant functions known to be effective in the healing process.

VR technologies can be used in health care to prepare children for procedures. Studies have shown that children’s VR techniques effectively prevent fear and stress in the health field. Increasing VR research in recent years includes studies on specific stress points such as anesthesia and vascular access applications.^[24–27]^ Large devices such as ECHO, MRI, CT, and ultrasound, as well as specially designed rooms where children experience severe stress, are needed, and there is a need for research on diagnostic procedures. In the literature, VR is used in preparation for MRI scans,^[28,29]^ VR preparation for CT procedures,^[30]^ and VR use in abdominal and heart ultrasound preparation^[31,32]^ studies.

These studies are important in using VR techniques, but they still need to be supported by multidimensional studies on the oxidative aspect of medical stress. Pediatric cardiology research still needs VR preparation and oxidative perspective for ECHO. This research can contribute to the literature.

## 5. Conclusion

3D film stress, developed for stress management in diagnosing and treating heart diseases in preparation for ECHO, causes a decrease in oxidative stress parameters at the cellular level. At the same time, the increased effect on the antioxidant factor shows that 3D film can be used effectively in ECHO. In ECHO, which has an important place in diagnosis in pediatric cardiology, 3D movies can be recommended for rapid and effective preparation of children as a precaution against the risks associated with sedation.

## 6. Strengths and limitations

Our research results covered 3 months prospectively according to the research inclusion criteria, and the data is limited to the sample. The study used routine blood from the children, and no additional blood was taken. In this process, blood samples that did not meet the criteria were excluded from the study. The study’s strength is that it presents new data to the literature by revealing the oxidative stress changes of the VR and ECHO preparation, which is applied for the first time in pediatric cardiology. In addition to the effect of the VR method on stress responses, the results will also contribute to the literature with GSH level changes to evaluate its impact on recovery. In the study, children were blinded to the registrar, research pediatric cardiologist, and biochemist who chose the experimental control group.

## Acknowledgments

This study was supported by Mustafa Kemal University Scientific Research Project Commission (Project No. 18.M.084). We would kindly thank the doctors, nurses, children, and their families who contributed to the 3D filming of the research.

## Author contributions

**Conceptualization:** Emel Demir.

**Data curation:** Nuh Yilmaz.

**Formal analysis:** Vesile Düzgüner, Erhan Yengil.

**Investigation:** Emel Demir, Nuh Yilmaz.

**Methodology:** Emel Demir, Vesile Düzgüner.

**Writing – original draft:** Emel Demir.

**Writing – review & editing:** Emel Demir, Vesile Düzgüner.
